# A clinico-statistical study of factors associated with intraoperative bleeding in orthognathic surgery

**DOI:** 10.1186/s40902-022-00336-8

**Published:** 2022-02-25

**Authors:** Keisuke Sugahara, Yu Koyama, Masahide Koyachi, Akira Watanabe, Kiyohiro Kasahara, Masayuki Takano, Akira Katakura

**Affiliations:** 1 Department of Oral Pathobiological Science and Surgery, Tokyo Dental College, 2-9-18 Kanda Misaki-cho, Chiyoda-ku, Tokyo, 101-0061 Japan; 2 Oral Health Science Center, Tokyo Dental College, 2-9-18 Kanda Misaki-cho, Chiyoda-ku, Tokyo, 101-0061 Japan; 3 Department of Oral and Maxillofacial Surgery, Tokyo Dental College, 2-9-18 Kanda Misaki-cho, Chiyoda-ku, Tokyo, 101-0061 Japan

**Keywords:** Orthognathic surgery, Risk factors, Intraoperative bleeding, Surgical planning, Bimaxillary surgery

## Abstract

**Background:**

Excessive bleeding is a major intraoperative risk associated with orthognathic surgery. This study aimed to investigate the factors involved in massive bleeding during orthognathic surgeries so that safe surgeries can be performed. Patients (*n*=213) diagnosed with jaw deformities and treated with bimaxillary orthognathic surgery (Le Fort I osteotomy and bilateral sagittal split ramus osteotomy) in the Department of Oral and Maxillofacial Surgery at the Suidobashi Hospital, Tokyo Dental College between January 2014 and December 2016 were included. Using the patients’ medical and operative records, the number of cases according to sex, age at the time of surgery, body mass index (BMI), circulating blood volume, diagnosis of maxillary deformity, direction of maxillary movement, operative duration, incidence of bad split, injury of nasal mucosa, and blood type were analyzed.

**Results:**

The results revealed that BMI, circulating blood volume, nasal mucosal injury, and operative time were associated with the risk of intraoperative massive bleeding in orthognathic surgeries. Chi-square tests and binomial logistic regression analyses showed significant differences in BMI, circulating blood volume, direction of maxillary movement, operative duration, and injury to the nasal mucosa. Operative duration emerged as the most important risk factor. Furthermore, a >4-mm upward migration of the posterior nasal spine predicted the risk of massive bleeding in orthognathic surgery.

**Conclusions:**

The upward movement of the maxilla should be recognized during the preoperative planning stage as a risk factor for intraoperative bleeding, and avoiding damage to the nasal mucosa should be considered a requirement for surgeons to prevent massive bleeding during surgery.

## Background

Owing to the recent development of surgical instruments and improvements in surgical procedures that have led to increased safety, orthognathic surgery is now a widely performed and important specialization within oral and maxillofacial surgery. With the inclusion of surgical orthodontic treatment in the Japan’s National Health Insurance coverage in 1990 and its increased social recognition, orthognathic surgery is now performed in many Japanese institutions [1, 2]. Orthognathic surgery is often performed on patients who are in good health, and it is important to perform the surgery with a greater emphasis on safety. In addition, due to changes in the esthetic and functional requirements of patients, more precise surgery is required. Therefore, a variety of surgical instruments and methods as well as surgical support software and devices based on digital technology have been actively developed [3–5].

The incidence of intraoperative and postoperative complications in orthognathic surgery is reported to be 6.1–9.0% [6–11]. In particular, major bleeding related to the patient’s general condition has been reported to occur in 0.085–1.1% of patients. Studies that report maxillary osteoplasty (Le Fort type I osteotomy) alone [12–15] have been previously performed, and a case of massive bleeding requiring ligation of the external carotid artery has been reported [13]. The causes of massive bleeding in orthognathic surgery include injury to the facial artery, maxillary artery, inferior alveolar artery, sublingual artery, posterior mandibular vein, and pterygopalatine venous plexus in mandibular surgery, and injury to the pterygopalatine venous plexus, maxillary artery, and descending palatine artery in maxillary surgery. However, the risk factors involved in intraoperative bleeding during orthognathic surgeries have not yet been investigated in detail.

A detailed assessment of bleeding risk at the time of surgery is important to ensure a safe surgery. In addition, preoperative evaluation of bleeding risk is expected to contribute to safe surgical planning, improvement of surgical methods, and development of devices. This study aimed to investigate the predictors of massive bleeding in 213 cases of maxillary and mandibular orthognathic surgeries performed at a single institution. The study’s findings could help in the treatment planning for safer surgical procedures.

## Methods

A total of 213 patients diagnosed with jaw deformities who were treated with bimaxillary orthognathic surgery (Le Fort I osteotomy [LFI] and bilateral sagittal split ramus osteotomy [BSSRO]) in the Department of Oral and Maxillofacial Surgery at the Tokyo Dental College, Suidobashi Hospital, between January 2014 and December 2016 were enrolled in the study. All cases of cleft lip and palate or multi-segmental osteotomy were excluded. The records of patients who underwent surgery as an intervention for malocclusion were examined.

### Analyzed factors

Using the patients’ medical and operative records, the number of cases according to sex, age at the time of surgery, body mass index (BMI), circulating blood volume, diagnosis of maxillary deformity, direction of maxillary movement, operative duration, bad split, injury to the nasal mucosa, and blood type were analyzed.

The patients were divided into two groups according to their age at the time of surgery: those who were 27 years or older, which was the average age of all 213 patients, and those younger than 27 years. A BMI≥25 was defined as obesity using the standard set by the Japan Society for the Study of Obesity, and patients with a BMI < 25 were classified as having a standard body shape. Normal circulating blood volume was defined as 70 g/kg. The body weight was calculated using the average Japanese body weight (60 kg for men and 50 kg for women) from the National Health and Nutrition Survey (70 g × 60+50/2 = 3850 g), and the subjects were divided into two groups. Clinical diagnoses of maxillary prognathism, maxillary retraction, and facial asymmetry were compared among the three groups. The upward movement of the posterior nasal spine (PNS) could be a potential risk for bleeding because it requires bone removal around the pterygopalatine fossa and descending palatine artery; therefore, patients were grouped based on whether the extent of upward migration of their PNS was <4 mm or ≥4 mm. Patients were also divided into two groups based on the duration of surgery, with a duration >4 h being considered a long surgery. The descending palatine artery was preserved in all cases in this study. Nasal mucosal injuries in this study were defined as those caused by perioperative laceration. And no turbinectomy was performed in any case. The method of calculating the intraoperative blood loss in this study was the sum of the weights of the suction cage and gauze minus the saline solution used. The surgeries reviewed in this study were performed under uniform and general anesthesia conditions.

### Statistical analysis

In this study, intraoperative massive bleeding group (459 g) was defined as the amount of blood loss exceeding 1 standard deviation (SD) from the mean blood loss over 3 years (279 g), and the chi-square test was used to compare the proportion of massive bleeding cases in each group. The predictors of massive bleeding extracted from chi-square tests were compared using binomial logistic regression analysis (ANOVA). Differences were considered statistically significant at *p* < 0.05. All data were processed using the IBM SPSS software package ver. 23. (Chicago, IL, USA).

## Results

**Fig. 1:**
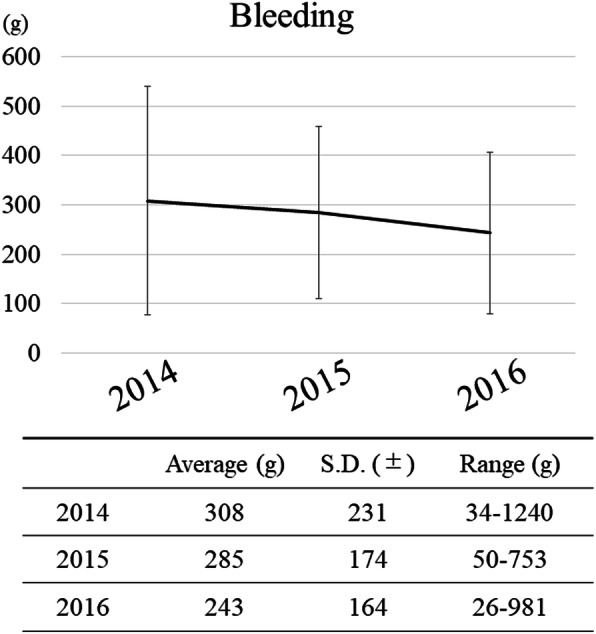
Annual change in bleeding volume

A total of 213 patients diagnosed with jaw deformities were treated with orthognathic surgery between January 2014 and December 2016. The annual trends of intraoperative blood loss during this period were 308 g, 285 g, and 243 g. The annual trend of blood loss decreased over time but was not significantly different, with a minimum of 26 g and a maximum of 1240 g, and a mean blood loss of 279 g overall (Fig. 1). The number of cases exceeding 1 SD from the mean blood loss (≥459 g) was 31 (14.6%). The percentage of massive bleeding and results of the chi-square test for each study item are shown in Table 1.

**Table 1:** Results of the chi-square test

Variables		All patients, no. (%)	Patients, no. (%)		*P* value
			Bleeding<459 g	Bleeding≧459 g	
Sex	Male	77 (36.2%)	61 (79.2%)	16 (20.8%)	0.053
	Female	136 (63.8%)	121 (89%)	15 (11%)	
Age	≧27	94 (44.1%)	85 (90.4%)	9 (9.6%)	0.067
	27>	119 (55.9%)	97 (81.5%)	22 (18.5%)	
BMI	≧25	13 (6.1%)	8 (61.5%)	5 (38.5%)	0.012
	25>	200 (93.9%)	174 (87%)	26 (13%)	
Circulating blood volume	≧3850 g	82 (38.5%)	65 (79.3%)	17 (20.7%)	0.043
	3850 g>	131 (61.5%)	117 (89.3%)	14 (10.7%)	
Diagnosis of maxillary deformity	Maxillary prognathism	23 (10.8%)	18 (78.3%)	5 (21.7%)	0.387
	Maxillary retrusion	72 (33.8%)	60 (83.3%)	12 (16.7%)	
	Facial asymmetry	118 (55.4%)	104 (88.1%)	14 (11.9%)	
Direction of maxillary movement	PNS≧4mm	10 (4.7%)	6 (60%)	4 (40%)	0.019
	4mm>PNS	203 (95.7%)	176 (86.7%)	27 (13.3%)	
Operative duration	≧4 h	105 (49.3%)	79 (75.2%)	26 (24.8%)	0.001
	4 h>	108 (50.7%)	103 (95.4%)	5 (4.6%)	
Bad split	Yes	5 (2.3%)	4 (80%)	1 (20%)	0.727
	No	208 (97.7%)	178 (85.6%)	30 (14.4%)	
Injury of nasal mucosa	Yes	86 (40.4%)	67 (77.9%)	19 (22.1%)	0.01
	No	127 (59.6%)	115 (90.6%)	12 (9.4%)	
Blood type	Type O	64 (30%)	54 (84.4%)	10 (15.6%)	0.771
	Exclusive of type O	149 (70%)	128 (85.9%)	21 (14.1%)	

### Number of cases by sex

Compared to men, more women (men: *n*=77, 36.2%; female: *n*=136, 63.8%) underwent orthognathic surgery (male to female, 1:1.8). Major bleeding occurred in 15 men (19.5%) and 16 women (11.8%). No statistically significant differences were found between groups.

### Number of cases by age at the time of surgery

The youngest patient was 16 years old, and the oldest was 52 years old, with an average age of 27 years. Age at the time of surgery was compared between patients older than 27 years (9/94 [9.6%]) and those younger than 27 years (22/119 [18.5%]). No statistically significant differences were found between groups.

### Number of cases by BMI and circulating blood volume

To evaluate the difference in intraoperative blood loss due to body size, BMI and estimated circulating blood volume of the 213 patients were compared. Thirteen patients (6.1%) were classified into the obese group. The rate of intraoperative massive bleeding in the obese group (5/13 cases [38.5%]) was significantly higher than that in the standard group (26/200 cases [13.0%]). The percentage of patients with a circulating blood volume of 3850 g or more (17/82 [20.7%]) was significantly higher than that of patients with a circulating blood volume of less than 3850 g (14/131 [10.7%]). There were statistically significant differences in BMI and circulating blood volume between the groups.

### Number of cases by diagnosis of maxillary deformity and direction of maxillary movement

To investigate whether the surgical technique affects intraoperative bleeding, the clinical diagnosis of the maxilla and the direction of movement were compared. The clinical diagnosis was facial asymmetry in 118 cases (55.4%), maxillary retraction in 72 cases (33.8%), and maxillary prognathism in 23 cases (10.8%). There was no significant difference in the rate of massive bleeding among the three groups. The extent of maxillary movement caused a significant difference between the groups, and an upward movement of 4 mm or more was found to increase the risk of bleeding.

### Number of cases by operative duration

**Fig. 2:**
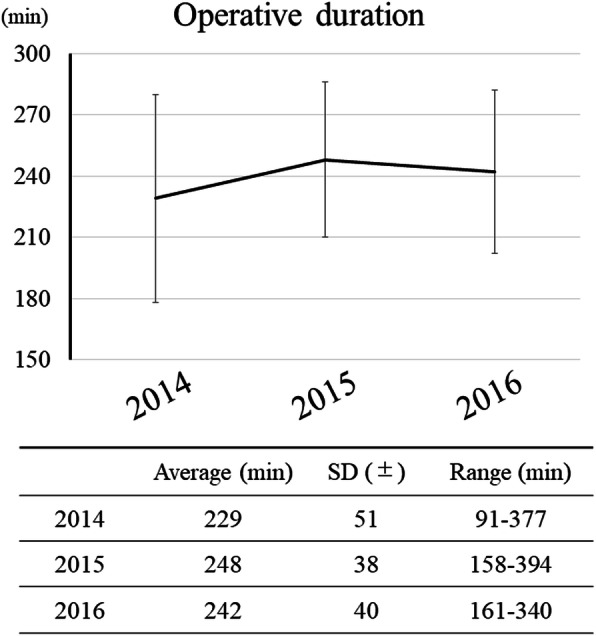
Annual change in operation duration

The mean operation time remained unchanged at 229, 248, and 242 min over the 3-year period, with the shortest operation time at 91 min and the longest at 391 min (Fig. 2). The study investigated whether the duration of surgery affected the amount of blood loss and found that the rate of intraoperative massive bleeding was significantly higher in the long surgery group (26/105 [24.8%]) than in the short surgery group (5/108 [4.6%]).

### Number of cases by incidence of bad split and injury to the nasal mucosa

Bad split and nasal mucosal injury, which are intraoperative complications of the down-fracture technique, were found in 5 (2.3%) and 86 patients (40.3%), respectively. There was no significant difference in the incidence of bad splits between the two groups. The nasal mucosal injury group (19/86 [22.1%]) had a significantly higher rate of massive bleeding than the non-injury group (12/127 [9.4%]).

### Number of cases by blood type

Since patients with blood type O are reported to have a higher risk of upper gastrointestinal bleeding [16], the rate of massive bleeding was compared between type O and other blood types. There were no statistically significant differences between the groups.

To further investigate whether the factors that showed significant differences in the chi-square test were associated with the risk of massive bleeding, a binomial logistic regression analysis was conducted using BMI, circulating blood volume, direction of maxillary movement, operative duration, and injury to the nasal mucosa as dependent variables and the presence of bleeding of 459 g or more as an independent variable (Table 2). There were significant differences in all the factors (Table 2).

**Table 2:** Results of the binomial logistic regression analysis

	OR	95% CI	*P* value
BMI	4.02	0.95–17.06	0.012
Circulating blood volume	1.15	0.38–3.54	0.043
Direction of movement	4.07	0.89–18.60	0.019
Operative duration	6.44	2.29–18.09	0.001
Injury of nasal mucosa	2.66	1.12–6.36	0.010

## Discussion

The most common causes of intraoperative bleeding during orthognathic surgeries are injury to the descending palatine artery, fracture of the pterygoid process, and major anatomic irregularities. Methods to control bleeding and avoid blood transfusion include (1) proficiency in orthognathic surgical techniques to ensure safe and reliable surgery and shorten operative duration, (2) use of local anesthesia containing epinephrine and application of hypotensive anesthesia [17], and (3) preparation of preoperative stored autologous blood. Katagiri et al. reported that the average operating time in Japan was 285 min (120–451 min) for maxillomandibular cases, and the average blood loss was 305 mL (32–872 mL) for maxillomandibular cases [1]. A review of 27 years of orthognathic surgeries from 1990 to 2017 at Suidobashi Hospital showed that the operative time decreased slightly from an average of 249 min (155–579 min) during 1990–2003 to an average of 233 min (85–484 min) during 2004–2017 [2]. In contrast, the amount of blood loss decreased dramatically from 899 g during 1990–2003 to 302 g during 2004–2017 [2]. In the present study, the reported operative time was slightly shorter than that reported by other institutions (249 min, 281 min, 290 min, 285 min). In addition, the amount of blood loss tended to be less than that reported by the Japanese Society for the Study of Jaw Deformities (512 g, 305 g) [1].

The rate of massive bleeding as a surgical complication is reported to be 0.05–11% [6–11]. Nonetheless, the need for blood transfusion to correct anemia caused by intraoperative bleeding during orthognathic surgery has been debated. In a previous report, the average blood loss in upper and lower jaw osteotomy was 889 g, and blood transfusion was recommended to prevent anemia [13]. However, the amount of intraoperative blood loss has decreased in recent years due to the development of surgical methods, general anesthesia, and devices. In Suidobashi Hospital, there were three cases (1.4%) with bleeding of more than 1000 g [2]. The bleeding was caused by injury of the descending palatal and inferior alveolar arteries. No cases of surgical interruption were reported by Zaroni et al [11]. All patients were asked for autogenous blood donation 6–8 weeks before surgery in Suidobashi Hospital. In the past, 400–800 mL of the autologous blood was usually collected from patients undergoing bimaxillary surgery, but the use of autologous blood varies, and there is no clear standard. In recent years, due to the decrease in overall intraoperative blood loss during most orthognathic surgeries, except in complex operations such as multifractional LFI, the use of the autologous blood is now at the surgeon’s discretion and it depends on the direction of jaw movement. This is due to the application of hypotensive anesthesia, ultrasonic cutting instruments, and developments in three-dimensional (3D) technology over the past decade, which have popularized the use of 3D printers in several institutions, especially in orthognathic surgeries. The “Fab Lab TDC” was the first digital fabrication laboratory for dentistry in Japan, which was established in 2013 [18]. Various 3D devices have been reported previously [5, 19].

Massive bleeding is a serious complication associated with dissection of the pterygoid maxillary suture in LFI [20]. To reduce this risk, the authors have been performing down-fractures using the leverage technique without dissecting the pterygoid maxillary suture with pterygomyxel [21]. In this method, the bone spreading Tessier forceps are inserted into the lateral border of the pterygomaxilla to open it up and then moved to the thick bone area near the inferior ridge of the cheekbone to push it downward to cause a “down-fracture.” The posterior part of the maxilla is then pushed downward with the Rowe forceps to achieve full mobility. It is theorized that the relatively small number of massive bleeding cases is due to these efforts. Kramer et al .[13] reported that 11 out of 1000 patients had massive bleeding, and 6 (5.2%) had major anatomic irregularities. Thus, if possible, contrast-enhanced computed tomography should be used to confirm the location of the blood vessels in the soft tissue before selecting a surgical technique and considering the direction of movement of the jawbone.

## Conclusions

In conclusion, this study aimed to analyze the factors associated with major bleeding during bimaxillary orthognathic surgery performed in a single center. All surgeries were performed under standardized general anesthesia conditions for the duration of the study. Factors related to bleeding that were independent of the type of general anesthesia were identified. The results revealed that BMI, circulating blood volume, nasal mucosal injury, and operative time were associated with the risk of intraoperative massive bleeding in orthognathic surgeries. In addition, the rate of intraoperative massive bleeding increased with statistical significance in patients with a ≥4-mm upward migration of the PNS. Based on the study’s findings, it is suggested that the upward movement of the maxilla should be recognized during the preoperative planning stage as a risk factor for intraoperative bleeding and that avoiding damage to the nasal mucosa should be considered a requirement for surgeons to prevent massive bleeding during surgery. Improvements in this area can only be achieved through an in-depth analysis of all procedures. Further studies with a large number of cases should be conducted, to aid orthognathic surgeons in achieving better results and safety.

## Data Availability

The analyzed data sets generated during the present study are available from the corresponding author on reasonable request.

## Abbreviations

BMI: Body mass index
LFI: Le Fort I osteotomy
PNS: Posterior nasal spine
SD: Standard deviation
3D: Three-dimensional
